# Clinical factors affecting functioning in patients with schizophrenia or schizoaffective disorder

**DOI:** 10.1192/j.eurpsy.2022.2015

**Published:** 2022-09-01

**Authors:** C. Neily, N. Charfi, G. Smaoui, N. Feki, R. Omri, L. Zouari, J. Ben Thabet, M. Maâlejboauli, M. Maalej

**Affiliations:** Hedi Chaker hospital, Psychiatry Department, Sfax, Tunisia

**Keywords:** functioning, schizophrénia

## Abstract

**Introduction:**

Schizophrenia is often associated with impaired functioning abilities due to its disabling symptoms.

**Objectives:**

to determine the clinical factors that impact the functioning in stabilized patients withschizophrenia and schizoaffective disorder.

**Methods:**

We conducted a cross-sectional, descriptive and analytical study. It was carried out on an outpatient population with schizophrenia or schizoaffective disorder diagnosis. We used the Functional Assessment Staging Scale (FAST) to measure the functional capacity, the PANSS to assess psychosis symptom severity and the Calgary scale to screen for comorbid depression.

**Results:**

Seventy-five patients were included with 61 males (81.3%).The mean age was 39.81 ± 9.96 years. The mean sore of the Fast scale was 33 ± 14.95. 90% of our patients scored higher than 11 on the FAST scale revealing a functioning deficiency. 18.7% scored higher than 6 on the Calgary scale revealing a comorbid depression .No significant correlations were found between the FAST score and the age of patient, the gender,the age of onset of psychosis, the duration of untreated psychosis and the number of life-time episodes. Scores of PANSS were significantly higher among patients with a functioning deficiency (p<0.00).No significant correlation was found between the FAST score and the Calgary score.

**Conclusions:**

Our study suggests that the severity of residual positive and negative symptoms affects negatively the functioning of patients with schizophrenia or schizoaffective disorder. Thus, targeting those symptoms in the treatment may have significant functional benefits.

**Disclosure:**

No significant relationships.

